# Spatial Navigation and Its Association With Biomarkers and Future Dementia in Memory Clinic Patients Without Dementia

**DOI:** 10.1212/WNL.0000000000201106

**Published:** 2022-11-08

**Authors:** Gro Gujord Tangen, Maria H. Nilsson, Erik Stomrud, Sebastian Palmqvist, Oskar Hansson

**Affiliations:** From the The Norwegian National Centre for Ageing and Health (G.G.T.), Vestfold Hospital Trust, Oslo ; Department of Geriatric Medicine (G.G.T.), Oslo University Hospital, Norway; Department of Health Sciences (M.H.N.), Lund Universityl Memory Clinic (M.H.N., E.S., S.P., O.H.), Skåne University Hospital, Malmö; and Clinical Memory Research Unit (M.H.N., E.S., S.P., O.H.), Department of Clinical Sciences Malmö, Lund University, Sweden.

## Abstract

**Background and Objectives:**

Impaired spatial navigation is considered an early sign in many neurodegenerative diseases. We aimed to determine whether spatial navigation was associated with future dementia in patients with subjective cognitive decline (SCD) or mild cognitive impairment (MCI) and to explore associations between spatial navigation and biomarkers of Alzheimer disease (AD) and neurodegeneration.

**Methods:**

This study included memory clinic patients without dementia in the longitudinal BioFINDER cohort. The Floor Maze Test (FMT) was used to assess spatial navigation at baseline. Conversion to dementia was evaluated at 2-year and 4-year follow-ups. At baseline, amyloid-β 42/40 ratio, phosphorylated-tau (P-tau), and neurofilament light (NfL) were analyzed in CSF. Cortical thickness and volume of regions relevant for navigation and white matter lesion volume were quantified from MRI. The predictive role of the FMT for conversion to all-cause dementia was analyzed using logistic regression analyses in 2 models: (1) controlled for age, sex, and education and (2) adding baseline cognitive status and MMSE. Associations between FMT and biomarkers were adjusted for age, sex, and cognitive status (SCD or MCI).

**Results:**

One hundred fifty-six patients with SCD and 176 patients with MCI were included. FMT total time was associated with progression to all-cause dementia in model 2 at 2-year (OR 1.10, 95% CI 1.04–1.16) and at 4-year follow-up (OR 1.10, 95% CI 1.04–1.16), i.e., a 10% increase in odds of developing dementia per every 10 seconds increase in FMT. In the adjusted analyses, P-tau and NfL were associated with FMT total time, as well as hippocampal volume, parahippocampal, and inferior parietal cortical thickness. Amyloid-β 42/40 ratio was not associated with FMT total time.

**Discussion:**

Impaired spatial navigation was associated with conversion to dementia within 2 and 4 years and with key CSF and MRI biomarkers for AD and neurodegeneration in patients with SCD and MCI. This supports its use in early cognitive assessments, but the predictive accuracy should be validated in other cohorts.

**Classification of Evidence:**

This is a Class I prospective cohort study demonstrating association of baseline markers of spatial recognition with development of dementia in patients with SCD or MCI at baseline.

A major neurocognitive disorder (i.e., dementia) is most often preceded by prodromal stages of subjective cognitive decline (SCD) and mild cognitive impairment (MCI). However, not all persons with SCD and MCI proceed to develop dementia, and it is therefore important to be able to detect those at most risk of subsequent dementia. Impaired spatial navigation is considered an early symptom of dementia and is also observed in persons with MCI.^1-4^ Studies on spatial navigation in SCD are few and with conflicting results.^2,5^ Impairments in spatial navigation are traditionally closely linked to the integrity of structures in the medial temporal lobe, which is the area where the first cell loss occurs in the development of Alzheimer disease (AD).^6^ However, spatial impairments are also observed in patients with other dementia subtypes such as dementia with Lewy bodies (DLB)^7,8^ and vascular dementia (VaD).^9,10^ Impairments in spatial navigation might offer a possibility for early identification of persons likely to subsequently develop dementia. This warrants the need for assessment tools that can identify impairments in spatial navigation at an early stage. Several virtual reality setups have been developed for this purpose; however, these are not readily available in clinical practice. An alternative approach is performance-based field tests. The Floor Maze test (FMT) was developed as a clinical feasible test of spatial navigation while walking, especially targeting the allocentric (maplike) navigation, which is dependent on the hippocampal regions.^11^ However, performance on the FMT has later also been linked closely with executive function.^1,12^

Our primary aim was to investigate the association between spatial navigation while walking, measured by the FMT, and future development of all-cause dementia in patients with SCD or MCI. Our secondary aims were (1) to study whether FMT was associated with progression to AD dementia and (2) to describe the associations between performance on FMT and *APOE ε4* status, CSF biomarkers for AD and neurodegeneration, and MRI measures of atrophy and cerebrovascular disease.

## Methods

### BioFINDER Study Population

This study is part of the larger longitudinal Swedish BioFINDER study (Biomarkers for Identifying Neurodegenerative Disorders Early and Reliably) as previously described.^13,14^ In summary, memory clinic patients were consecutively recruited if they fulfilled these inclusion criteria: (1) referred because of cognitive symptoms experienced by the patient and/or informant, (2) between ages 60 and 80 years, (3) Mini-Mental Status Examination (MMSE)^15^ score between 24 and 30 points, (4) fluent in Swedish, and (5) did not fulfill the criteria for dementia. Exclusion criteria were (1) cognitive impairment at baseline that could be explained by another condition or disease (e.g., brain tumor), (2) significant unstable systemic illness making participation difficult, (3) current significant alcohol or substance abuse, or (4) refusing lumbar puncture or neuropsychological assessment. They were classified as either SCD or MCI based on the performance of a comprehensive neuropsychological battery with 2 tests for each of the 4 cognitive domains: verbal ability, episodic memory, visuospatial ability, and attention and executive function. Patients with a mean z-score ≤ −1.5 (relative to normative score) in at least 1 cognitive domain were classified as having MCI.^14^ The patients with a z-score between −1.0 and −1.5 were also classified as MCI if their performance was believed to represent a substantial decline from premorbid cognitive function, based on an individual assessment by a neuropsychologist. The SCD classification was used for patients who did not fulfill the MCI criteria because they all had subjective concerns for their cognitive function leading to seeking medical help.^16^ At the 2-year and 4-year follow-up, the *DSM-5* criteria for major neurocognitive disorder (dementia) due to probable AD were used to diagnose AD. In addition, the patient was required to show signs of abnormal amyloid accumulation according either to CSF analysis^17^ or to Aβ PET^13^ in agreement with the National Institute on Aging–Alzheimer's Association criteria for AD.^18^ The respective *DSM-5* criteria were used also for the diagnosis of all-cause dementia, including DLB, frontotemporal dementia, and VaD. These diagnoses were set based on the treating physician's follow-up assessments and reviewed by a consensus group including memory clinic physicians and a senior neuropsychologist. Diagnoses were assigned without information about the results on the FMT. More details of the study design and diagnostic procedures can be found at the website^19^ and in previous publications.^13,14^

This study had additional inclusion/exclusion criteria which determined the final sample size; the participant should have performed the FMT during the assessment by a physical therapist at the memory clinic, and 6 participants who used walking aids indoors were also excluded. Only participants recruited from Skåne University Hospital had FMT assessments. We also included data from the 2-year and 4-year follow-ups, where the patients were assessed by a physician, including cognitive tests, and collecting information from informants on cognitive and daily functioning using standardized scales.

### Standard Protocol Approvals, Registrations, and Patient Consents

The study was approved by the Regional Ethical Review Board in Lund, Sweden. All participants gave their written informed consent to participate.

### Assessment of Spatial Navigation

The FMT was used to assess spatial navigation while walking.^11^ The FMT is a 2-dimensional 7 × 10 ft maze made of dark gray carpet with white tape indicting the lines of the maze (Figure). The physical therapist positioned the patient at the entry of the maze and instructed them to find their way to the exit of the maze. Performance was timed, including time to plan how to solve the maze (planning time [PT]) and the time of walking through the maze (immediate maze time [IMT]). Our main outcome was FMT total time, which is the combined time of PT and the IMT. The original FMT included delayed maze time (DMT)—a second walk through the maze, 10 minutes after the first walk.^11^ We chose to only include the IMT, and the median time to administer this modified version of the FMT was 1 minute, although for the slowest participants, the test was finished after 5 minutes.

**Figure:**
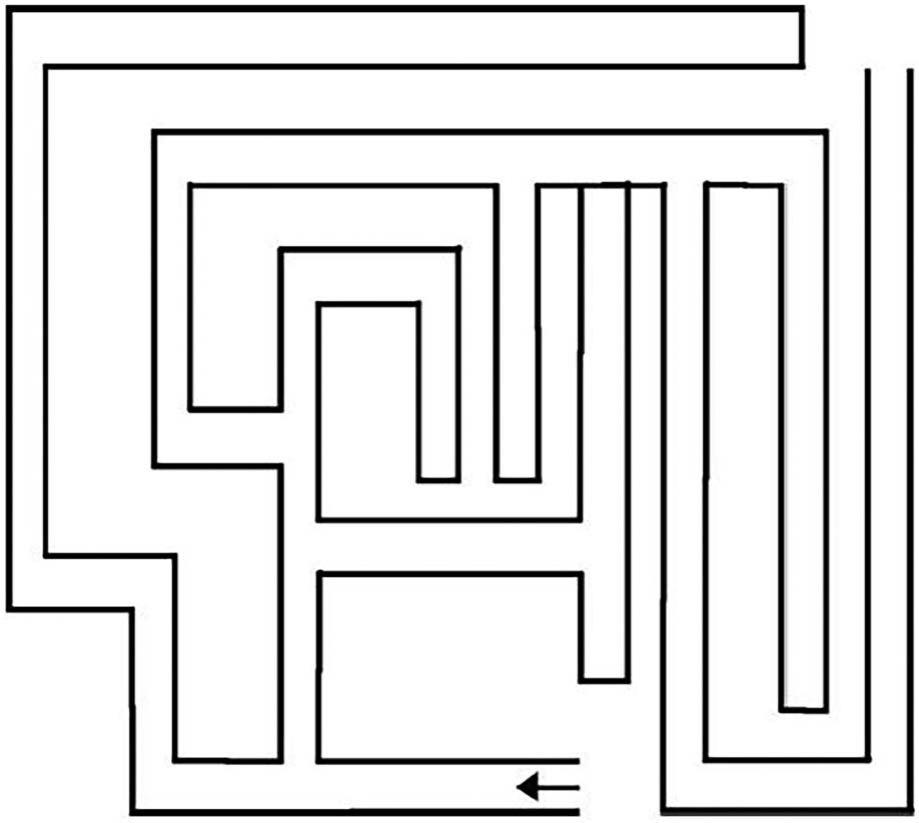
The Floor Maze Test Figure from International Psychogeriatrics.^1^ Reproduced with permission from Cambridge University Press.

In addition, patients were categorized into “error-free” vs “with error” (any wrong turn in the maze or crossing of lines) performance and into those who completed vs not completed (tried but gave up either during planning or during the walk) the maze. The FMT assessments were videotaped with a standardized video recording protocol for research purposes. Timing and categorization of performances were performed based on the video tapes. The 2 raters (G.G.T., M.H.N.) were blinded for cognitive test results, biomarker results, and cognitive status when rating. The interrater reliability and test-retest reliability for the FMT has not been established; however, the correlation coefficient between the IMT and the DMT in another study was *r* = 0.76.^11^

### Biomarkers

Lumbar puncture and handling of CSF samples followed a structured protocol as described in a previous publication.^13^ CSF amyloid-β (Aβ) 40 and Aβ42 were analyzed by EUROIMMUN ELISAs (EUROIMMUN AG Lübeck, Germany). Pathologic Aβ accumulation was considered as present using a previously established CSF Aβ42/40 cutoff <0.088,^20^ i.e., determined by using mixture modeling statistics in the healthy controls and participants with SCD and MCI in BioFINDER.^21,22^ Tau phosphorylated at Thr181 (P-tau) were analyzed with INNOTEST ELISA (Fujirebio Gent, Belgium). Neurofilament light (NfL) was measured using a commercial ELISA at the Clinical Neurochemistry Laboratory at University of Gothenburg, Sweden (NF-light ELISA; Uman Diagnostics, Umeå, Sweden).^23^

### Magnetic Resonance Imaging

High-resolution, T1-weighted magnetization-prepared rapid gradient-echo (repetition time = 1950 ms, echo time = 3.4 ms, in-plane resolution = 1 × 1 mm^2^, slice thickness = 1.2 mm, 176 slices) and T2-weighted fluid-attenuated inversion recovery (repetition time = 9,000 ms, echo time = 89 ms, T1 = 2,500 ms, voxel size 0.7 × 0.7 × 4 mm^3^, distance factor 25%, 27 slices) imaging was performed on the same 3T MR scanner (Siemens Tim Trio 3T; Siemens Medical Solutions, Erlangen, Germany) for all participants. The FreeSurfer image analysis pipeline v5.3^24^ was used for cortical reconstruction and volumetric segmentation, as described in previous publication.^25^ We excluded MRI data from 11 participants because of low quality.

To avoid too many comparisons, we focused on mean values of regions well-known for their role in spatial navigation; hippocampal volume, and thickness of entorhinal, parahippocampal, inferior parietal, precuneus, posterior cingulate cortices, and the prefrontal cortex.^26^ Mean cortical thickness (left and right) for the prefrontal cortex was obtained by merging the following structures (not weighted for surface area): superior frontal gyrus, middle frontal gyrus (rostral division), inferior frontal gyrus (pars opercularis, pars orbitalis, pars triangularis), orbitofrontal cortex (lateral and medial division), frontal pole, and the anterior cingulate cortex (rostral and caudal division).^27^ Global white matter lesion (WML) volume was established using an automated segmentation method, applying the lesion prediction algorithm in the lesion segmentation toolbox toolbox implemented in SPM8.^28^

### Statistical Analysis

The main outcome in this study was subsequent development of all-cause dementia within (1) 2-year or (2) 4-year follow-up. Secondary outcome was progression to AD dementia within (1) 2-year or (2) 4-year follow-up. As exploratory outcomes, we studied associations between performance on FMT and CSF biomarkers and MRI. Comparisons of characteristics between the SCD and the MCI groups were performed using the Student *t* test or the Mann-Whitney *U* test depending on distribution and the χ^2^ test for categorical variables (Table 1).

**Table 1:**
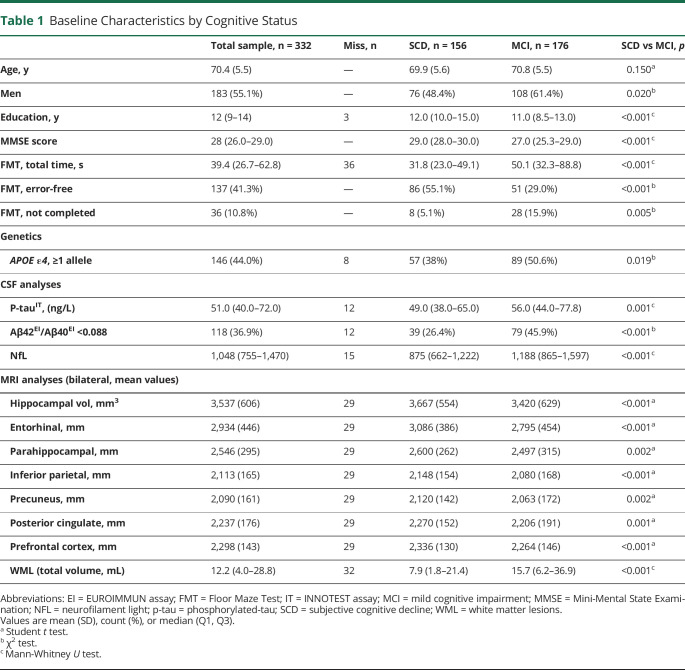
Baseline Characteristics by Cognitive Status

The predictive role of the 3 FMT outcomes was analyzed using logistic regression analyses, with conversion to all-cause dementia (coded as 1) vs nonconversion (coded 0) as dependent variable (Table 2). Two models were used: model 1 regression analyses with age, sex, and education and model 2 adding baseline cognitive status (SCD or MCI) and MMSE. Three participants did not have data on education and were not part of these analyses. For sensitivity analyses, we also report the associations for conversion to all-cause dementia adjusted for age, sex, education, and MMSE stratified by baseline SCD and MCI status. For 36 participants who did not complete the FMT, their result on FMT total time was imputed as the time of the slowest finisher (i.e., 254 seconds). To obtain clinical meaningful information, we converted the FMT total time into 10-second increments in line with a previous publication.^12^ For sensitivity purposes, the analyses were also performed without imputation on FMT total time. We also performed the same statistical procedures with conversion to AD dementia as the dependent variable (Table 3).

**Table 2:**
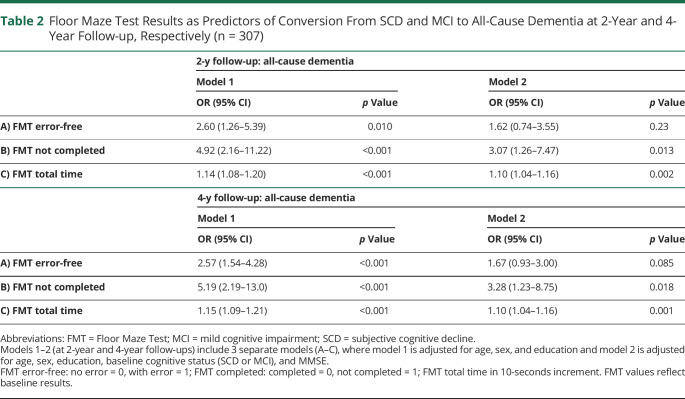
Floor Maze Test Results as Predictors of Conversion From SCD and MCI to All-Cause Dementia at 2-Year and 4-Year Follow-up, Respectively (n = 307)

**Table 3:**
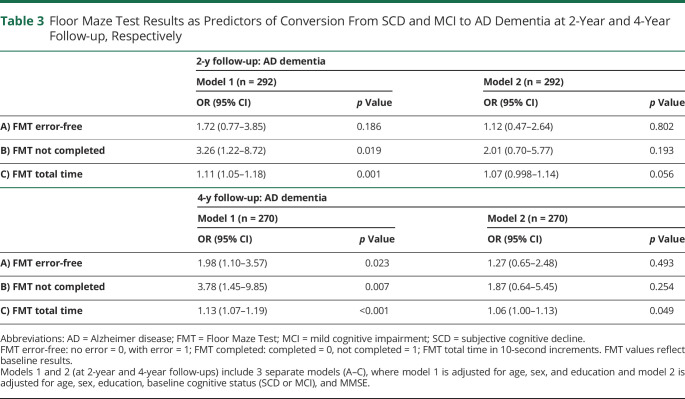
Floor Maze Test Results as Predictors of Conversion From SCD and MCI to AD Dementia at 2-Year and 4-Year Follow-up, Respectively

To test the power of the FMT total time to discriminate between converters and nonconverters, the area under the receiver operating characteristics curve (AUC) was assessed, and the sensitivity and specificity was calculated at the cutoff yielding the highest Youden index (Table 4). Comparison of the accuracy of the FMT, MMSE, and a combination of the FMT and MMSE were performed using logistic regression models with these tests as independent variables and conversion to all-cause dementia or AD dementia dependent variables. Comparisons of AUCs were performed using DeLong statistics. These analyses were performed in R version 4.0; all other analyses were performed using IBM SPSS Statistics version 27.

**Table 4:**
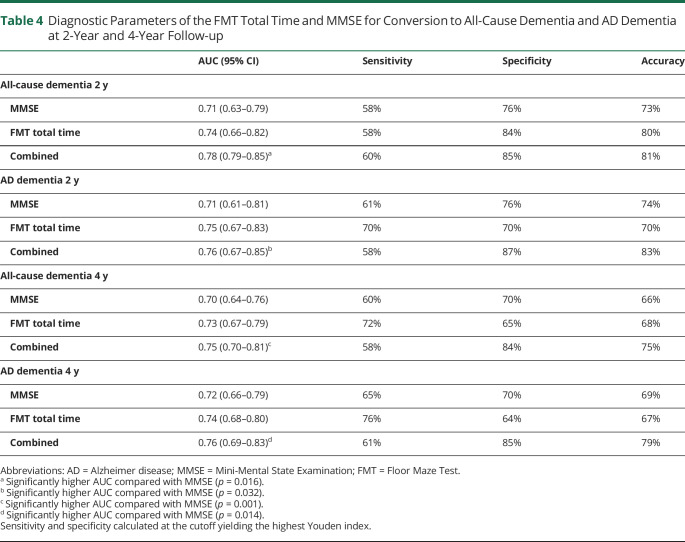
Diagnostic Parameters of the FMT Total Time and MMSE for Conversion to All-Cause Dementia and AD Dementia at 2-Year and 4-Year Follow-up

The associations between FMT and key biomarkers, *APOE ε4* genotype (1 or 2 ε4 alleles vs no ε4 alleles), CSF Aβ42/40 (Aβ pathology), CSF P-tau (tau pathology), CSF NfL (neurodegeneration), cortical thickness of regions of interest (parietal lobe, medial temporal lobe, and prefrontal cortex), hippocampal volume, and WML volume (cerebral small vessel disease) at baseline were analyzed in 2 types of models: (1) univariate regression analyses and (2) regression analyses with each biomarker separately controlled for age, sex, and baseline cognitive status (SCD or MCI) (Table 5). For models with hippocampal volume, we also included intracranial volume as a covariate.

**Table 5:**
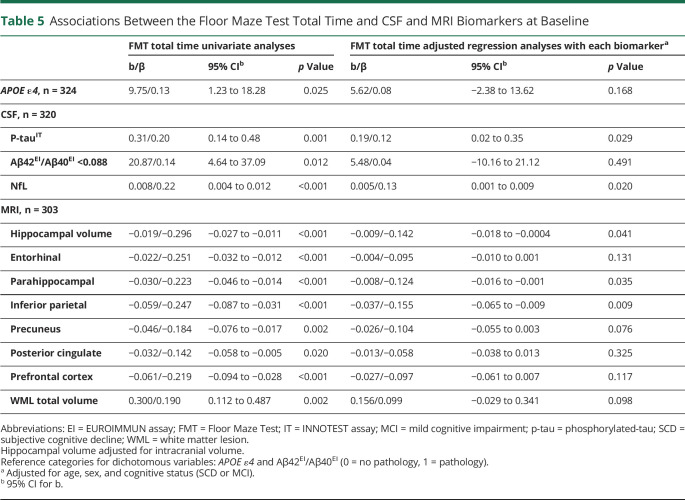
Associations Between the Floor Maze Test Total Time and CSF and MRI Biomarkers at Baseline

### Data Availability

Anonymized data will be shared by request from a qualified academic investigator for the sole purpose of replicating procedures and results presented in the article and as long as data transfer is in agreement with EU legislation on the general data protection regulation and decisions by the Swedish Ethical Review Authority and Region Skåne, which should be regulated in a material transfer agreement.

## Results

The sample for this study (n = 332) included 156 patients with SCD and 176 patients with MCI at baseline (Table 1). Participants with MCI had lower education than the participants with SCD (*p* < 0.001), and there were more men in the MCI group (*p* = 0.02). There were statistically significant differences between the SCD and MCI groups on the cognitive assessments, all biomarkers, and on the FMT outcomes in favor of the SCD group.

### FMT as a Predictor of Conversion to All-Cause Dementia

Of the 332 participants included at baseline, 310 (93.4%) had valid data on dementia status at the 2-year and 4-year follow-up. Among these, 48 (15.2%) had developed dementia after 2 years and 112 participants (35.4%) after 4 years. All FMT outcomes were significantly associated with conversion to all-cause dementia at both 2-year and 4-year follow-up in the simple adjusted logistic regression models (including age, sex, education). In the next model adding baseline cognitive status and MMSE, FMT “total time” and “not completed” were significantly associated with conversion to dementia both within 2-year and 4-year (Table 2) follow-up. The OR (95% CI) for FMT total time at 2-year follow-up was 1.10 (1.04–1.16) and at 4-year follow-up was 1.10 (1.04–1.16), indicating that there is a 10% increase in odds of developing dementia per every 10 seconds increase.

In the analyses stratified by baseline SCD and MCI status, FMT total time was significantly associated with conversion to dementia at 2-year follow-up: OR (95% CI) was 1.24 (1.02–1.50, *p* = 0.035) for SCD vs 1.08 (1.02–1.15, *p* = 0.012) for MCI. At 4-year follow-up, FMT total time was significantly associated with all-cause dementia in the SCD group with OR (95% CI) at 1.28 (1.12–1.46. *p* < 0.001), which was not the case for the MCI group with OR (95% CI) at 1.04 (0.98–1.10, *p* = 0.25). In the sensitivity analyses without imputation (n = 276), FMT total time was significantly associated with conversion both at 2-year follow-up (OR 1.17, 95% CI 1.07–1.28, *p* = 0.001) and 4-year follow-up (OR 1.19, 95% CI 1.09–1.29, *p* < 0.001) in model 1. In model 2, FMT total time was no longer associated with conversion at 2-year follow-up (OR 1.01, 95% CI 0.996–1.22, *p* = 0.60); at the 4-year follow-up, the OR was 1.09 (1.00–1.19, *p* = 0.05). The AUC (95% CI) of the FMT total time, MMSE, and for the FMT and MMSE combined is presented in Table 4. Adding FMT to the MMSE increased the accuracy for identifying converters to all-cause dementia from 73% to 81% at 2-year follow-up and from 66% to 75% at 4-year follow-up.

### FMT as a Predictor of Conversion to AD Dementia

At the 2-year follow-up, 33 patients (10.4%) were diagnosed with AD dementia, 7 (2.2%) with VaD, and 4 (1.3%) with DLB/PDD and with FTD. Corresponding numbers after 4 years were 74 (23.4%) with AD, 21 (6.7%) with VaD, 10 (3.2%) with DLB/PDD, 4 (1.3%) with FTD, and 3 (1.0%) with other dementia. We repeated the analyses of conversion, with conversion to AD dementia instead of all-cause dementia at 2-year and 4-year follow-up as the dependent variable. In these analyses, FMT total time and FMT not completed were significantly associated with conversion to AD dementia in the minimally adjusted models including age, sex, and education at the 2-year follow-up, while all FMT outcomes were associated with conversion at the 4-year follow-up (Table 3). In model 2, none of the FMT variables were significantly associated with conversion to AD dementia at 2-year follow-up, while FMT total time was significantly associated with conversion to AD dementia, OR (95% CI) 1.06 (1.00–1.13, *p* = 0.049) at the 4-year follow-up. Adding FMT to the MMSE increased the accuracy for identifying converters to AD dementia from 74% to 83% at 2-year follow-up and from 69% to 79% at 4-year follow-up. The AUCs for the combined MMSE and FMT were significantly higher compared with MMSE for all 4 end points (*p* = 0.001–0.032).

In the sensitivity analyses without imputation (n = 280), FMT total time was significantly associated with conversion to AD dementia in the minimally adjusted models including age, sex, and education at 2-year follow-up (OR 1.15, 95% CI 1.04–1.28, *p* = 0.007) and 4-year follow-up (OR 1.17, 95% CI 1.07–1.27, *p* = 0.001). In the models including baseline cognitive status and MMSE, FMT total time was no longer associated with conversion to AD dementia at neither 2-year (OR 1.08, 95% CI 0.97–1.21, *p* = 0.16) nor at 4-year follow-up (OR 1.08, 95% CI 0.98–1.19, *p* = 0.11).

### Associations Between FMT Total Time, *APOE ε4*, and CSF Biomarkers for AD and Neurodegeneration at Baseline

In the univariate analyses, each of the CSF biomarkers and *APOE ε4* status were significantly associated with FMT total time (all *p* < 0.05) (Table 5). In the analyses adjusted for age, sex, and cognitive status at baseline, increased level of CSF P-tau (*p* = 0.03) and NfL (*p* = 0.02), but not Aβ42/40 ratio (*p* = 0.49) nor *APOE ε4* status (*p* = 0.17), were associated with FMT total time.

### Associations Between FMT Total Time and MRI Measures of Atrophy and WMLs

Each region of interest was significantly associated with FMT total time in the univariate analyses (all *p* < 0.005) (Table 5). In the adjusted analyses, hippocampal volume (adjusted for intracranial volume) (*p* = 0.041), inferior parietal (*p* = 0.009), and thickness of parahippocampal (*p* = 0.035) cortex remained significant, while the other regions and WML total volume were no longer significantly associated with FMT total time (all *p* > 0.05).

### Classification of Evidence

This is a Class I prospective cohort study demonstrating association of baseline markers of spatial recognition with development of dementia in patients with SCD or MCI at baseline.

## Discussion

The main finding of this study is that spatial navigation during walking, measured using the FMT, was associated with conversion to dementia at 2-year and 4-year follow-up in patients with SCD and MCI at baseline. Its association with conversion to specifically AD dementia was less robust than conversion to all-cause dementia, indicating that other neurodegenerative disorders also have early impaired spatial navigation. At baseline, worse performance on the FMT was associated with pathology in CSF biomarkers, especially P-tau and NfL, and with less hippocampal volume and reduced thickness in parahippocampal and inferior parietal cortices.

To identify those who will develop dementia among community-dwelling persons with subtle cognitive complaints is a challenging task that entails a demanding diagnostic workup with access to advanced assessment of biomarkers from blood, CSF, or brain imaging. Although this approach to dementia diagnostics is available in the specialist health care in high-income countries, assessments in less-resourceful settings may rely on less advanced approaches. Spatial navigation is a complex cognitive task relevant for all persons regardless of age and socioeconomic factors. Using virtual reality to assess spatial navigation has its obvious benefits regarding flexibility to develop tasks with high specificity in surroundings mimicking real-life. However, such equipment will likely be predominantly available in the same settings as the advanced dementia diagnostics. It is therefore imperative to develop other approaches which use the potential for using assessment of spatial navigation as a cognitive biomarker to identify persons with increased risk of developing subsequent dementia.

The FMT total time was associated with progression to all-cause dementia at both 2-year and 4-year follow-up in our study. Our results are in line with findings from the Einstein Aging Study, where performance on the FMT predicted development of MCI and motoric cognitive risk syndrome (MCR) in a cohort of community-dwelling older adults without dementia, MCI, or MCR at baseline.^12^ There are also several cross-sectional studies reporting worse performance on the FMT with increasing severity of cognitive impairment.^1,29,30^

Spatial navigation has been brought to attention in research focusing on predementia groups given the location of place cells,^31^ grid cells,^32^ and head direction cells^33^ in the same brain regions as where AD pathology is first observed.^34^ Thus, one should have believed that the association between FMT and progression to AD dementia should be stronger than for all-cause dementia. While the FMT total time was associated with development of AD dementia in the simple model 1 at both 2-year and 4-year follow-up, this association remained significant only at 4-year follow-up in model 2 also including baseline cognitive status and MMSE. Our apparently paradoxical findings can have several explanations. First, lack of power because there is obviously lower sample size for a specific dementia subtype, and the association at 2-year follow-up is rather close to statistically significant (*p* = 0.056). However, given that the AD group was as large as n = 33 at the 2-year follow-up, it should be large enough to detect a clinical important association. In addition, we aimed to focus on all-cause dementia rather than AD dementia. This was to a large extent based on clinical experience, having conversations also with patients with non-AD dementia talking about their increasing problems with wayfinding in both familiar and unfamiliar surroundings even in the early stage of dementia. As stated in the introduction, there is emerging evidence from other studies that also persons with DLB and VaD have impaired spatial navigation abilities.^7–10^ Our results further concur with the findings from the Einstein Aging Study, where the association between FMT and incident MCI was driven by nonamnestic MCI and not by amnestic MCI (aMCI).^12^ Their finding was supported by subanalyses in which FMT predicted decline in executive function, but not progression of memory impairment.

The value of examining spatial cognition for prediction of progression of cognitive impairment was also illustrated in a proof-of-concept study including 15 patients with MCI.^35^ The authors used the 4 Mountains Test to examine allocentric spatial memory, and the test predicted conversion from MCI to AD dementia with 93% accuracy. In addition, in a heterogeneous group of 95 participants with cognitive functioning ranging from cognitively normal to mild symptomatic AD, route learning performance predicted clinical progression and discriminated between progressors and nonprogressors.^36^ Future studies will be needed to determine whether the promising results for these outcomes are valid in larger cohorts. The AUC of the FMT for conversion to all-cause dementia and AD at 2-year and 4-year follow-up was fair at 0.73–0.75 in our study which has a lot larger sample than the previously mentioned studies. In addition, the combined AUC for MMSE and FMT was significantly higher than for MMSE alone, indicating that the FMT contributes with additional prognostic information that can be of clinical importance.

Another frequently used nonvirtual test protocol is the Hidden Goal Task, a human analog of the Morris water maze, which examines egocentric and allocentric navigational strategies.^37^ Few studies have used a real-life setting, although there are examples such as the Route learning test where the participants are taken in a wheelchair through a hospital environment.^38^ The FMT is however one of the few tests that includes walking and turning, which may contribute to its ecological validity. Common findings across these different test paradigms and the FMT are that performance is worse in patients with cognitive impairment compared with cognitively healthy older adults^30,37,38^ and comparable associations with dementia pathology as outlined below.

The important role of spatial navigation for identification of concurrent dementia pathology is underlined by our findings of significant associations between the FMT and each of the CSF biomarkers in the unadjusted analyses. After controlling for age, sex, and cognitive status, the associations with P-tau and NfL remained significant. While P-tau is an AD-specific biomarker,^39^ NfL is marker of general neuronal damage.^40^ Previous studies have reported that persons with aMCI who are *APOE ε4* allele carriers have worse performance on spatial navigation tasks compared with noncarriers.^41,42^ Others have reported that cognitively unimpaired persons with CSF levels of Aβ42 < 500 pg/mL (preclinical AD) had deficits in aspects of wayfinding compared with cognitively unimpaired persons without pathologic levels of Aβ42.^43^ In line with this result, patients with aMCI with pathologic levels of Aβ have shown worse performance on both egocentric and allocentric navigation tasks than those without Aβ pathology.^4^ Our study expands this knowledge by examining the associations between spatial navigation and a broader set of dementia biomarkers.

We also observed significant associations between FMT and each of the chosen brain regions known to be important for spatial navigation in the unadjusted analyses. Hippocampal volume as well as parahippocampal and inferior parietal cortex thickness remained significantly associated with the FMT also in the analyses adjusted for age, sex, and cognitive status. These associations support the validity of the FMT as a measure of spatial navigation. It is important to remember that human navigation does rely on a large network of brain regions, also beyond the hippocampal region.^26,44^ Given the executive component of the FMT, we assumed that the prefrontal cortex would also be associated with the FMT. Although there was a significant association in the bivariate analyses, this association was no longer significant in the adjusted analysis. Taken together, our findings indicate that impairments in spatial navigation performance in the predementia stage are related to biomarkers representing disease severity.

Our study had limitations. First, while other studies using the FMT have reported the original PT and IMT, we chose to combine these 2 outcomes into FMT total time. Our decision was based on the observation of some patients being very careful planners and have a long PT combined with a shorter IMT. Others almost skip planning, jumping to the first solution, and end up spending a long time on the IMT. Thus, the combined time of PT and IMT serves as a better representation of how the patients perform on this task. The FMT protocol has been further developed after our data collection by standardizing the PT to 15 seconds for all.^12^ Second, in the sensitivity analyses without imputation on the FMT total time for those who did not complete the test, the FMT did not significantly predict conversion to dementia or AD dementia in the adjusted models. However, we think it is very important to include these patients in the analyses because they all had given their best effort without succeeding, likely representing those who had the most severe impairments in spatial navigation. Strengths of the study include the large, well-described study cohort with a very low loss to follow-up even after 4 years, the broad selection of dementia biomarkers, and the use of a spatial navigation task that is easily feasible even in low-income countries.

In conclusion, FMT results at baseline were associated with subsequent conversion to all-cause dementia at 2-year and 4-year follow-up and to AD dementia at 4-year follow-up. Future studies are needed to explore spatial navigation performance across different dementia subtypes and in populations beyond memory clinic patients, as well as establish the predictive ability to identify patients getting lost. The FMT was also associated with established biomarkers for dementia and neural correlates well-known for their role for spatial navigation. Our findings indicate that assessment of spatial navigation can be included in memory clinics and primary care setting.

## Glossary

AD: Alzheimer disease
aMCI: amnestic MCI
AUC: area under the curve
DLB: dementia with Lewy bodies
DMT: delayed maze time
*DSM*: *Diagnostic and Statistical Manual of Mental Disorders*
FMT: Floor Maze test
IMT: immediate maze time
MCI: mild cognitive impairment
MMSE: Mini-Mental Status Examination
NfL: neurofilament light
PT: planning time
P-tau: phosphorylated-tau
SCD: subjective cognitive decline
VaD: vascular dementia
WML: white matter lesion
